# Examining tobacco prevention and cessation among dental students in Southern Italy

**DOI:** 10.1186/s12982-026-01569-y

**Published:** 2026-03-26

**Authors:** Olga Di Fede, Samuel Tundealao, David Okuji, Gaetano La Mantia, Irene Tamí-Maury

**Affiliations:** 1 Department of Precision Medicine in Medical, Surgical and Critical Care (Me.Pre.C.C.), University of Palermo, Palermo, Italy; 2 Department of Biostatistics and Epidemiology, Rutgers School of Public Health, Piscataway, NJ USA; 3 Harvard School of Dental Medicine, Cambridge, MA USA; 4 Unit of Oral Medicine and Dentistry for Fragile Patients, Department of Rehabilitation, Fragility and Continuity of Care, University Hospital Palermo, Palermo, Italy; 5 Department of Biomedical and Dental Sciences and Morphofunctional Imaging, University of Messina, Messina, Italy; 6 Department of Epidemiology, School of Public Health, The University of Texas Health Science Center at Houston, 1200 Pressler Street, Suite E641, Houston, TX 77030 USA

**Keywords:** Counseling, Dental students, ENDS, Public health, Tobacco cessation, Tobacco use

## Abstract

**Background:**

Previous studies have reported that tobacco use among healthcare providers (HCPs) has a negative impact on their beliefs and attitudes toward tobacco prevention and control measures. However, little is known about these among Italian dental students. Therefore, this single-site study examined tobacco use behaviors and the knowledge, attitudes, and perceptions of dental students at a public university in Southern Italy regarding tobacco prevention and control.

**Methods:**

A cross-sectional study through an online survey was conducted among 115 students aged 18 + years enrolled in dentistry and dental hygiene programs at a Southern Italy university. Chi-squared, Fisher's, *t*-tests, and multivariable logistic regression were used to compare the socio-demographic characteristics, tobacco use, prevention and cessation knowledge, attitudes, and perceptions between dentistry and dental hygiene students.

**Results:**

Current prevalence of cigarette smoking, electronic nicotine delivery systems (ENDS), and other tobacco product use among students was 30.4% (CI: 27.3–33.4), 24.4% (CI: 21.7–28.6), and 16.5% (CI: 12.9–19.2), respectively. There were no significant differences in tobacco use by student type after adjustment for socio-demographic characteristics. About 38% were using at least one form of tobacco product, while 27% were dual users of cigarettes and ENDS products. The prevalence of second-hand smoking, ENDS vapes, and other tobacco product exposure among dental students was 65.2%, 36.5%, and 20.9%, respectively. Around 55% disagreed that HCPs who smoke are less likely to advise patients to quit smoking. Dental students demonstrated greater knowledge on cigarette smoking danger (87.0%), reasons why people smoke (63.5%), and received formal training in cigarette smoking cessation approaches (68.7%) versus their knowledge/motivations/training related to ENDS and other tobacco products.

**Conclusion:**

The normalization of tobacco use (including ENDS products) among dental students engaged in our study is deeply concerning and highlights the need for targeted tobacco prevention and cessation strategies in dental education, while further multicenter studies are required to assess whether similar patterns exist in other Italian dental schools.

## Introduction

Cigarette smoking and consumption of other tobacco products, including chewed tobacco and/or electronic nicotine delivery systems (ENDS), pose a significant threat to public health, affecting virtually every organ in the body, triggering numerous diseases, and undermining the overall well-being of users [1–3]. In high-income countries, tobacco use remains a leading cause of mortality and morbidity. Despite the implementation of numerous tobacco control interventions worldwide, tobacco consumption remains a significant global public health challenge, contributing to significant economic and societal burdens [4, 5].

Healthcare professionals, including dentists and dental hygienists, play a crucial role in providing information about the harms of cigarette smoking and encouraging smoking cessation among patients [6]. Their expertise in oral health, close patient relationships, high frequency of appointments, and ability to identify smoking-related symptoms and clinical conditions in oral hard and soft tissues make them ideal advocates for tobacco cessation interventions [7].

Considering that tobacco use contributes significantly to major oral health issues such as oral cancer, periodontal disease, caries, and halitosis, among other oral conditions, dentists and dental hygienists emerge as frontline healthcare professionals in the battle against cigarette smoking and the use of other types of tobacco products, including ENDS [8–10]. Campaigns aimed at strengthening the knowledge, skills, and attitudes of oral health professionals toward tobacco cessation are therefore critical for ensuring their effectiveness in patient counseling and preventive care. It is conflicting with public health objectives when oral health professionals engage in tobacco use themselves or possess limited knowledge of cessation strategies, as this undermines both their credibility and their ability to act as role models for their patients. In addition, the World Health Organization (WHO) has issued policy recommendations for incorporating brief tobacco interventions into oral health programs within primary care, in alignment with both the WHO Oral Health Program’s tobacco control policy and the WHO policy on tobacco cessation [11]. Similarly, the FDI World Dental Federation has developed a tobacco cessation guide specifically designed for oral health professionals [12].

A study conducted in Italy and Poland revealed that the prevalence of tobacco consumption is surprisingly high among dental students of both nationalities [7]. However, this issue takes on significantly more critical dimensions in Italy, involving 42% of Italian students compared to 28% of their Polish counterparts. Furthermore, the prevalence of smoking among dental students in Italy has increased by 10% compared to the study conducted by Pizzo et al. in 2010 [13]. According to La Torre, increased tobacco use is emerging among healthcare professionals. One of the reasons for this situation is the inadequate coverage of tobacco-related issues in the medical curriculum [14]. Students, in fact, seem not to receive sufficient training in counseling patients to quit smoking, or, as per Cattaruzza et al., even if they receive training, this aspect is not considered a priority [14]. Assisting patients in quitting smoking represents a form of prevention, but students primarily focus on treatment [6].

As dental students represent the future generation of oral health practitioners, it is important to recognize that their knowledge, attitudes, and behaviors concerning the use of tobacco products and nicotine addiction can exert a significant influence on tobacco prevention and control initiatives in the country. This extends to the dental faculty members responsible for effectively increasing the knowledge and awareness and developing the skills among their students for delivering effective tobacco prevention and cessation interventions in dental settings [15]. To date, the literature contains surveys that compare dental students from different countries. Nevertheless, recent studies exclusively targeting Italian dental students are lacking, and there is also a noticeable scarcity of recent research involving dental hygiene students and dental faculty members [16, 17]. In Italy, the dentistry program typically spans six years and prepares graduates to diagnose, treat, and manage a wide range of oral diseases and conditions. In contrast, the dental hygiene program generally lasts three years and focuses primarily on the prevention of oral diseases, patient education, and the promotion of oral health. While both professions play complementary roles within the oral healthcare system, these differences in training duration and professional functions are important to acknowledge when interpreting findings related to tobacco use and cessation practices among dental students.

Addressing this gap was the primary objective of our investigation. Therefore, the aim of this study is to investigate a range of factors associated with tobacco consumption among students enrolled in dental programs at an Italian academic institution, as well as the curriculum content and institutional policies that such dental school has on tobacco use and nicotine addiction.

## Methods

### Study design, site, and participants

A cross-sectional study based on an online survey was conducted from March 1 to July 15, 2023, in a convenience sample of students (≥ 18 years) enrolled in dentistry or dental hygiene programs at the University of Palermo Dental School (UPDS), Italy. Only students who were providing clinical care to patients were eligible to participate in the study.

Based on the total enrollment of 150 students in the Dentistry and Dental Hygiene programs at the University of Palermo and considering the reported incidence of tobacco/nicotine consumption of 17.8% among Italian healthcare workers [18], a minimum sample size of 91 students was determined to be necessary to adequately assess the landscape of tobacco use in this population. We recruited 115 dental students in the study. For the purposes of this study, 'dental students' refers collectively to both dentistry and dental hygiene students, unless otherwise specified.

A convenience sampling approach was used because it allowed for efficient data collection within the limited timeframe and resources available for the study. In addition, given that the total number of dental students was known (150) and our target enrollment was at least 91, the use of convenience sampling, combined with the voluntary nature of participation, was considered sufficient to yield a representative sample of the dental student population at the university.

### Data collection and management

Data were collected using a self-administered questionnaire which comprised of 38 questions and grouped into five sections—(1) demographic details, (2) tobacco use behaviors, (3) school policy on tobacco use, (4) knowledge and attitudes towards tobacco (cigarettes, ENDS, and other tobacco products), tobacco prevention, and cessation, and (5) tobacco in dental school training/curriculum. The questionnaire was adapted from a previous study that assessed the landscape of tobacco prevention and cessation among dental students in Latin American countries [19].

The online survey was built and managed on Qualtrics, an online data collection and managing platform that securely transmits research data. The online survey was pilot-tested among ten UPDS dental students, and relevant modifications were implemented before initiating data collection.

### Measures

#### Demographic characteristics

Information about age, current year of enrollment, student type (dentistry or dental hygiene), and sex at birth was obtained from the study participants. Dentistry students in Years I-II and dental hygiene students in Year I were referred to as pre-clinical students. Clinical students were dentistry students in Years III-VI and dental hygiene students in Years II-III. Six years is required to complete the dentistry program, while three years is required for the dental hygiene program at UPDS.

#### Tobacco use

We also collected data from participants about their use of (a) the conventional cigarette, (b) ENDS products (e.g., e-cigarettes, e-cigs, vapes, vaporizers, vape pens, etc.), and (c) other tobacco products (e.g., cigars, pipe, chewed tobacco, hookah/narghile/water pipe etc.). Information was also gathered regarding where UPDS students use these tobacco products, such as clinical settings (where dental patients are treated), other indoor environments (e.g., waiting rooms, classrooms, etc.), and outdoor environments (e.g., parking lots, sidewalks, etc.). Second-hand smoking, ENDS vape exposure, and willingness to stop using tobacco products were also assessed.

Current smokers were classified as those individuals who answered "Yes" to the question "Have you smoked at least 100 cigarettes in your lifetime?" and responded, "Every day and Some days" to the question "How frequently did you smoke in the last 30 days?" [20]. Former smokers were those who had smoked at least 100 cigarettes but had not smoked some days or every day in the last 30 days [20]. Ever smokers were defined as dental students who had smoked 100 or more cigarettes in their lifetime, while Never smokers were dental students who had not smoked up to 100 cigarettes in their lifetime [20]. "Former smokers" and "Never smokers" were subsequently combined as non-current smokers. Current ENDS smokers were those who have used a form of ENDS product in the last 30 days.

Current "Dual Users" were participants using both cigarettes and ENDS products concurrently. Current "All Users" were individuals using all forms of tobacco products (cigarettes + ENDS + Other tobacco products), while current "Any Users" were using at least one form of tobacco product (i.e., cigarettes or ENDS or other tobacco product).

#### Knowledge and attitude

Information concerning knowledge, perceptions, and attitudes towards tobacco, tobacco prevention, and cessation was obtained from the students.

#### Dental school training/curriculum

The data collection instrument also included questions about tobacco use, prevention, and intervention approaches in the UPDS curricula.

#### School policy

Data on UPDS students' awareness of a formal policy prohibiting tobacco use and its enforcement in their academic institution were also collected.

### Statistical analysis

The following statistical analyses were conducted.

- *Demographic Characteristics:* Descriptive statistics were used to summarize the demographic characteristics of the study participants.
- *Tobacco Use:* Chi-squared test (or Fisher exact test when appropriate) was used to compare the different tobacco-related variables between the dentistry and dental hygiene students.
- *Knowledge and Attitude:* Responses to the knowledge, perceptions, and attitudes were dichotomized (Yes *vs.* No/Don't know). The proportions were presented for each question.
- *Dental school training/curriculum:* The inclusion of tobacco use, prevention, and cessation in the UPDS curricula was compared between the dentistry and dental hygiene students using the Chi-squared test (or Fisher exact test when appropriate).
- *School Policy****:*** Participants' knowledge of existing tobacco policies at UPDS was summarized and compared between the two groups of students (dentistry *vs.* dental hygiene programs).

Multivariable logistic regression was conducted to examine the association between student type and tobacco product use. Three models were fitted, one for cigarette smoking, one for ENDS use, and the last one for other tobacco product use. Each model was adjusted for sociodemographic characteristics, including age, sex, year of study, tobacco knowledge, and attitudes of oral health professionals toward tobacco cessation. These covariates were selected a priori (a priori approach) based on established knowledge of demographic and tobacco-related factors that may influence the outcomes. The significance threshold was set at 0.05. All quantitative analyses were conducted using Stata v. 17.0/SE (StataCorp, College Station, Texas).

## Results

### Demographic characteristics

The demographic characteristics of the dental students are described in Table 1. Study participants included 72 (62.6%) dentistry students and 43 (37.4%) dental hygienist students, most of them females (61.7%) with an average age of 23.2 (± 2.94) years. Two-thirds of the participants (67.8%) were in the clinical stage of their dental training, while the remaining 32.1% were pre-clinical dental students.

**Table 1:** Demographic and tobacco use characteristics of the study participants

Characteristics	Total N = 115	Dental students 72 (62.6%)	Dental hygiene student 43 (37.4%)	*P*-value
Enrollment stage Pre-clinical^a^ Clinical^b^	37 (32.1%) 78 (67.8%)	22 (30.6%) 50 (69.4%)	15 (34.9%) 28 (65.1%)	0.313
Age (in years) mean ± SD	23.2 (± 2.94)	23.1 (± 2.17)	23.3 (± 3.9)	0.6354
Sex Male Female	44 (38.3%) 71 (61.7%)	34 (47.2%) 38 (52.8%)	10 (23.3%) 33 (76.7%)	**0.011**
Current smoking Yes No	35 (30.4%) 80 (69.6%)	23 (31.9%) 49 (68.1%)	12 (27.9%) 31 (72.1%)	0.649
Current use of ENDS products^c^ Yes No	28 (24.4%) 87 (75.6%)	16 (22.2%) 56 (77.8%)	12 (27.9%) 31 (72.1%)	0.492
Current use of other tobacco products^d^ Yes No	19 (16.5%) 96 (83.5%)	13 (18.1%) 59 (81.9%)	6 (13.9%) 37 (86.1%)	0.567
Current dual users (cigarette + ENDS) Yes No	31 (27.0%) 84 (73.0%)	20 (27.8%) 52 (72.2%)	11 (25.6%) 32 (74.4%)	0.797
Current all users (cigarette + ENDS + other) Yes No	7 (6.1%) 108 (93.9%)	3 (4.2%) 69 (95.8%)	4 (9.3%) 39 (90.7%)	0.265
Current any user (uses any tobacco product) Yes No	44 (38.3%) 71 (61.7%)	29 (49.3%) 43 (59.7%)	15 (34.9%) 28 (65.1%)	0.565
Witnessed tobacco product use in clinical settings over the past year Smoked cigarettes Yes No ENDS Yes No Other tobacco products Yes No	2 (1.7%) 113 (98.3%) 5 (4.4%) 110 (95.6%) 1 (0.9%) 114 (99.1%)	2 (2.8%) 70 (97.2%) 2 (2.8%) 70 (97.2%) 1 (1.4%) 71 (98.6%)	0 (0.0%) 43 (100.0%) 3 (7.0%) 40 (93.0%) 0 (0.0%) 43 (100.0%)	0.270 0.285 0.438
Witnessed tobacco product use in other indoor environments over the past year Smoked Cigarettes Yes No ENDS Yes No Other tobacco products Yes No	0 (0.0%) 115 (100.0%) 2 (1.7%) 113 (98.3%) 0 (0.0%) 115 (100.0%)	0 (0.0%) 72 (100.0%) 1 (1.4%) 71 (98.6%) 0 (0.0%) 72 (100.0%)	0 (0.0%) 43 (100.0%) 1 (2.3%) 42 (97.7%) 0 (0.0%) 43 (100.0%)	0.710 1.000 1.000
Witnessed tobacco product use in outdoor environments over the past year Smoked Cigarettes Yes No ENDS Yes No Other tobacco products Yes No	18 (15.7%) 97 (84.3%) 21 (18.3%) 94 (81.7%) 3 (2.6%) 112 (97.4%)	15 (20.8%) 57 (79.2%) 11 (15.3%) 61 (84.7%) 3 (4.2%) 69 (95.8%)	3 (7.0%) 40 (93.0%) 10 (23.3%) 33 (76.7%) 0 (0.0%) 43 (100.0%)	***0.048*** 0.284 0.175
Reporting last month exposure to Second-hand smoke Yes No ENDS aerosols Yes No Smoke or aerosols from other tobacco products Yes No	75 (65.2%) 40 (34.8%) 42 (36.5%) 73 (63.5%) 24 (20.9%) 91 (79.1%)	55 (76.4%) 17 (23.6%) 28 (38.9%) 44 (61.1%) 20 (27.8%) 52 (72.2%)	20 (46.5%) 23 (53.5%) 14 (32.6%) 29 (67.4%) 4 (9.3%) 39 (90.7%)	***0.001*** 0.495 ***0.019***
Wanting to quit Smoking Cigarettes (n = 35) Yes No Using ENDS (n = 28) Yes No Using other tobacco products (n = 19) Yes No	16 (45.7%) 19 (54.3%) 11 (39.3%) 17 (60.7%) 4 (21.1%) 15 (78.9%)	12 (52.2%) 11 (47.8%) 5 (31.3%) 11 (68.7%) 2 (15.4%) 11 (84.6%)	4 (33.3%) 8 (66.7%) 6 (50.0%) 6 (50.0%) 2 (33.3%) 4 (66.7%)	0.288 0.315 0.372
Attempted to quit during last year Smoking Cigarettes (n = 35) Yes No Using ENDS (n = 28) Yes No Using other tobacco products (n = 19) Yes No	13 (37.1%) 22 (62.9%) 7 (25.0%) 21 (75.0%) 2 (10.5%) 17 (89.5%)	10 (43.5%) 13 (56.5%) 2 (12.5%) 14 (87.5%) 2 (15.4%) 11 (84.6%)	3 (25.0%) 9 (75.0%) 5 (41.7%) 7 (58.3%) 0 (0.0%) 6 (100.0%)	0.283 0.103 0.310
Received help or advice for your previous attempts to quit Smoking Cigarettes (n = 35) Yes No Using ENDS (n = 28) Yes No Using other tobacco products (n = 19) Yes No	11 (31.4%) 24 (68.6%) 6 (21.4%) 22 (78.6%) 1 (5.3%) 18 (94.7%)	9 (39.1%) 14 (60.9%) 2 (12.5%) 14 (87.5%) 1 (7.6%) 12 (92.3%)	2 (16.7%) 10 (83.3%) 4 (33.3%) 8 (66.7%) 0 (0.0%) 6 (100.0%)	0.259 0.354 0.554

Bold italics = statistical significance (*p* < 0.05)

^a^ Years I and II for dentistry students, Year I for dental hygiene students

^b^ Years III, IV, V, and VI for dentistry students, Years II and III for dental hygiene students

^c^ Such as e-cigarettes, e-cigs, vapes, vaporizers, vape pens, JUULS, hookah pens, e-cigars, e-pipes

^d^ Such as cigars, pipe, chewed tobacco, hookah/narghile/water pipe, snuff, dip, snus

### Tobacco use

**Fig. 1:**
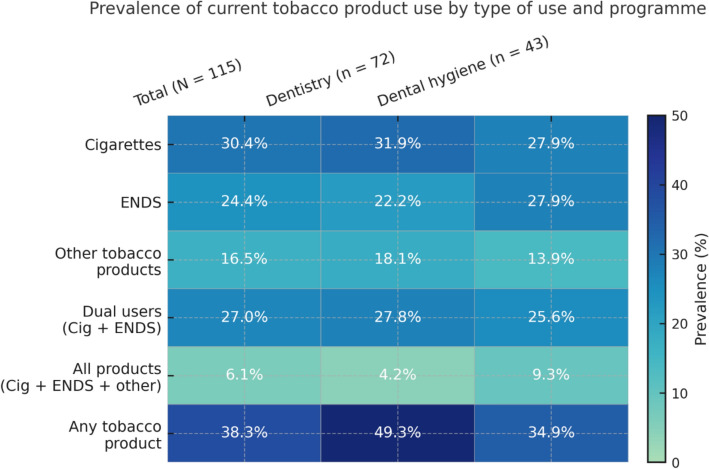
Heatmap of the prevalence of current use of tobacco products among the students enrolled

Approximately 4 in 10 (35%) dental students were ever smokers. The prevalence of current smoking among them was 30.4% (CI 27.3–33.4), indicating that 3 out of 10 dental students were current cigarette smokers. Half of the participants (50.4%) were ever ENDS users, while the prevalence of current ENDS use was 24.4% (CI 21.7–28.6). Among the dental students, 43.5% had used other tobacco products at one time in their lives. The current prevalence of other tobacco product use among the dental students was 16.5% (CI 12.9–19.2). About 38% were using at least one form of tobacco product (cigarette or ENDS or other tobacco product), 27% were dual users of cigarettes and ENDS, while 6% used all three forms of tobacco products. The prevalence of exposure to second-hand smoking, ENDS aerosoles, and aerosols from other tobacco products among dental students was 65.2%, 36.5%, and 20.9%, respectively. The willingness to quit was about 46% among current smokers, 41% among current ENDS users, and 21% among those who currently use other tobacco products. Detailed information about the students' tobacco use characteristics is in Table 1. Figure 1 also illustrates the prevalence of current use of cigarettes, ENDS, and other tobacco products by student type.

Approximately 1.7% of dental students reported observing individuals smoking cigarettes in clinical settings, while none reported such observations in the university’s indoor environment; however, 15.7% reported witnessing cigarette smoking in outdoor areas of the university. With respect to ENDS and other tobacco products, 4.4% and 0.9% of students, respectively, reported observing their use in clinical settings; 1.7% and 0% in indoor environments; and 18.3% and 2.6% in outdoor environments, respectively (Table 1).

There are statistically significant differences between exposure to second-hand smoke (76.4% vs. 46.5%; *p* = 0.001) and aerosols from other tobacco products (27.8% vs. 9.3%; *p* = 0.019) between dentistry and dental hygiene students, respectively (Table 1). There was no statistically significant difference between the tobacco-related variables and student type (cigarette use—aOR: 1.10, 95% CI: 0.91–1.23, *p* = 0.318; ENDS use—aOR: 0.98, 95% CI 0.96–1.05, *p* = 0.896, and other tobacco product—aOR: 1.19, 95% CI 0.68–1.77, *p* = 0.173) after adjusting for sociodemographic characteristics.

### Tobacco knowledge and attitudes

Dental students largely support tobacco control measures, including bans on selling tobacco to adolescents (89.6%) and advertisements (66.1%), as well as restrictions on smoking in public places (71.3%). They also believe that healthcare practitioners should actively advise patients to quit tobacco use and receive training for tobacco cessation (88.7%). In addition, 94.8% believe healthcare providers have a role to play in informing their patients about smoking cessation. However, while most students think healthcare professionals can positively influence patients' quitting habits, only 45.2% believe that practitioners who smoke or use tobacco themselves are less likely to offer advice (Table 2).

**Table 2:** Knowledge and attitudes of study participants towards tobacco, tobacco prevention, and cessation

Characteristics	Total N = 115	Dental students 72 (62.6%)	Dental hygiene student 43 (37.4%)	*P*-value
Tobacco sales to adolescents (persons younger than 18 years old) should be banned Yes No/don't know	103 (89.6%) 12 (10.4%)	67 (93.1%) 5 (6.9%)	36 (83.7%) 7 (16.3%)	0.113
There should be a complete ban on tobacco products advertisement Yes No/don't know	78 (66.1%) 39 (33.9%)	51 (70.8%) 21 (29.2%)	25 (58.1%) 18 (41.9%)	0.164
Cigarette smoking and ENDS use should be banned in discos/bars/pubs Yes No/don't know	79 (68.7%) 36 (31.3%)	51 (70.8%) 21 (29.2%)	28 (65.1%) 15 (34.9%)	0.552
Cigarette smoking and ENDS use should be banned in restaurants Yes No/don't know	104 (90.4%) 11 (9.6%)	63 (87.5%) 9 (12.5%)	41 (95.4%) 2 (4.6%)	0.166
Cigarette smoking and ENDS use should be banned in all enclosed public places Yes No/don't know	82 (71.3%) 33 (28.7%)	52 (72.2%) 20 (27.8%)	30 (69.8%) 13 (30.2%)	0.778
Health practitioners should routinely advise ENDS users to quit Yes No/don't know	102 (88.7%) 13 (11.3%)	64 (88.9%) 8 (11.1%)	38 (88.4%) 5 (11.6%)	0.933
Health practitioners should have specific training on cessation techniques Yes No/don't know	102 (88.7%) 13 (11.3%)	65 (90.3%) 7 (9.7%)	37 (86.1%) 6 (13.9%)	0.488
Health practitioners should routinely advise current cigarette smokers to quit Yes No/don't know	103 (89.6%) 12 (10.4%)	64 (88.9%) 8 (11.1%)	39 (90.7%) 4 (9.3%)	0.759
Health practitioners should routinely advise ENDS users to quit Yes No/don't know	102 (88.7%) 13 (11.3%)	64 (88.9%) 8 (11.1%)	38 (88.4%) 5 (11.6%)	0.933
Health practitioners should routinely advise patients who use other tobacco products to quit Yes No/don't know	106 (92.2%) 9 (7.8%)	68 (94.4%) 4 (5.6%)	38 (88.4%) 5 (11.6%)	0.291
Health practitioners have a role in advising patients or informing them about smoking cessation Yes No/don't know	109 (94.8%) 6 (5.2%)	68 (94.4%) 4 (5.6%)	41 (93.4%) 2 (4.6%)	0.833
The patient's chances of quitting smoking cigarettes or using ENDS increase if health practitioners advise them Yes No/don't know	83 (72.2%) 32 (27.8%)	52 (72.2%) 20 (27.8%)	31 (72.1%) 12 (27.9%)	0.988
Health practitioners who smoke cigarettes or use ENDS are less likely to advise patients to quit Yes No/don't know	52 (45.2%) 63 (54.8%)	29 (40.3%) 43 (59.7%)	23 (53.5%) 20 (46.5%)	0.168

### Dental school training/curriculum

Compared to ENDS and other tobacco products, the dental students were more knowledgeable on the dangers of cigarette smoking (87.0%), why people smoke cigarettes (63.5%), and the importance of including smoking history in the general medical history (93.9%). About 68.7% have received formal training in cigarette smoking cessation approaches. Although most dental students had heard of nicotine replacement therapy (80.9%), only 44.3% had heard about the antidepressants (e.g., Bupropion or Zyban) used in cigarette smoking cessation programs (Table 3). There was a statistical significance between dental students' type (dental student vs dental hygiene student) and the awareness of the use of antidepressants in cigarette smoking cessation programs (36.1% vs. 58.1%; *p* = 0.021), respectively.

**Table 3:** Tobacco prevention and control in UPDS curricula

Characteristics	Total N = 115	Dental students 72 (62.6%)	Dental hygiene student 43 (37.4%)	*P*-value
Were you taught in any of your classes during your dental school training, the dangers of. Cigarettes smoking Yes No ENDS use Yes No Other tobacco products use Yes No	100 (87.0%) 15 (13.0%) 52 (45.2%) 63 (54.8%) 79 (68.7%) 36 (31.3%)	66 (91.7%) 6 (8.3%) 31 (43.1%) 41 (56.9%) 52 (72.2%) 20 (27.8%)	34 (79.1%) 9 (20.9%) 21 (48.8%) 22 (51.2%) 27 (62.79%) 16 (37.2%)	0.052 0.547 0.291
During your dental school training, did you discuss in any of your classes, the reasons people… Smoke cigarettes Yes No Use ENDS products Yes No Use other tobacco products Yes No	73 (63.5%) 42 (36.5%) 44 (38.3%) 71 (61.7%) 53 (46.1%) 62 (53.9%)	46 (63.9%) 26 (36.1%) 26 (36.1%) 46 (63.9%) 30 (41.7%) 41 (58.3%)	27 (62.8%) 16 (37.2%) 18 (41.9%) 25 (58.1%) 23 (53.5%) 20 (46.5%)	0.906 0.539 0.218
During your dental school training, have you ever received any formal training in using tobacco cessation approaches with patients who. Smoke cigarettes Yes No Use ENDS products Yes No Use other tobacco products Yes No	79 (68.7%) 36 (31.3%) 44 (38.3%) 71 (61.7%) 51 (44.3%) 64 (55.7%)	49 (68.1%) 23 (31.9%) 25 (34.7%) 47 (65.3%) 27 (37.5%) 45 (62.5%)	30 (69.8%) 13 (30.2%) 19 (44.2%) 24 (55.8%) 24 (55.8%) 19 (44.2%)	0.848 0.312 0.056
During your dental school training, did you learn that it is important to record as part of the general medical history, the history of. Cigarettes smoking Yes No ENDS use Yes No Other tobacco products use Yes No	108 (93.9%) 7 (6.1%) 64 (55.7%) 51 (44.3%) 80 (69.6%) 35 (30.4%)	70 (97.2%) 2 (2.8%) 42 (58.3%) 30 (41.7%) 52 (72.2%) 20 (27.8%)	38 (88.4%) 5 (11.6%) 22 (51.2%) 21 (48.8%) 28 (65.1%) 15 (34.9%)	0.101 0.454 0.423
During your dental school training, did you learn that it is important to provide educational materials to support tobacco cessation to patients who want to stop/quit. Cigarettes smoking Yes No ENDS use Yes No Other tobacco products use Yes No	98 (85.2%) 17 (14.8%) 59 (51.3%) 56 (48.7%) 70 (60.9%) 45 (39.1%)	62 (86.1%) 10 (13.9%) 36 (50.0%) 36 (50.0%) 42 (58.3%) 30 (41.7%)	36 (83.7%) 7 (16.3%) 23 (53.5%) 20 (46.5%) 28 (65.1%) 15 (34.9%)	0.727 0.717 0.471
Have you ever heard of nicotine replacement therapy (e.g., nicotine patches or gums) in cigarette smoking cessation programs? Yes No/don't know	93 (80.9%) 22 (19.1%)	59 (81.9%) 13 (18.1%)	34 (79.1%) 9 (20.9%)	0.705
Have you ever heard of antidepressants (e.g., Bupropion or Zyban) in cigarette smoking cessation programs? Yes No/don't know	51 (44.3%) 64 (55.7%)	26 (36.1%) 46 (63.9%)	25 (58.1%) 18 (41.9%)	***0.021***

Bold italics = statistical significance (*p* < 0.05)

### School policy

Most dental students (87.8%) indicated that their dental school had an official policy banning tobacco use. Of these students, 72% believed the tobacco policy is enforced inside the school buildings and/or clinics. There is a statistically significant difference in the knowledge of the enforcement of the school tobacco policy between the dentistry and dental hygiene students (63.9% vs. 84.6%; *p* = 0.025), respectively. (Table 4).

**Table 4:** Knowledge of school tobacco policy among the dental students

Characteristics	Total N = 115	Dental students 72 (62.6%)	Dental hygiene student 43 (37.4%)	*P*-value
My dental school has an official policy banning tobacco use (including ENDS use) Yes No	101 (87.8%) 14 (12.2%)	62 (86.1%) 10 (13.9%)	39 (90.7%) 4 (9.3%)	0.565
The tobacco policy is enforced inside school buildings and/or clinics? (n = 101) Yes No	72 (72.0%) 28 (28.0%)	39 (63.9%) 22 (36.1%)	33 (84.6%) 6 (15.4%)	***0.025***

Bold italics = statistical significance (*p* < 0.05)

## Discussion

Dental practitioners, including dental students, can play a pivotal role in mitigating the prevalence of tobacco-related chronic diseases through the proactive implementation of tobacco prevention and cessation strategies among their patients [21]. However, the normalization of tobacco use and nicotine consumption within the dental care community poses a significant challenge, potentially undermining these efforts by negatively impacting practitioners' attitudes toward tobacco prevention and control measures [21]. Preliminary findings from this cross-sectional study conducted among students enrolled in dental programs at an academic institution in Southern Italy offer a valuable opportunity to not only assess students' prevalence of tobacco use but also to delve into their knowledge, attitudes, and perceptions regarding tobacco prevention and control.

The current smoking prevalence among the students participating in our study (30.4%) is greater than the average observed among the general population globally (17%), in 12 European nations (25.9%), and in Italy (18.9%) [22–24]. The prevalence of current ENDS use was found to be 40% higher among dental students compared to the general Italian population [25]. This higher prevalence among dental students may be related to stressors associated with academic workload and the university environment, which are known to influence tobacco use behaviors. Another possible explanation is the cultural normalization of tobacco use, particularly among young adults. From a behavioral psychology perspective, this normalization may reinforce social learning and reward-based cues that make ENDS use more appealing and habitual within peer networks. In addition, a sense of perceived invulnerability (which manifests as an underestimation of the long-term health risks associated with smoking, and even more so with vaping) may further contribute to this behavior. Gender and regional cultural factors may also contribute to these patterns, as tobacco and ENDS use in Southern Italy have been shown to be influenced by gender norms, social identity, and community-level acceptance of smoking [26]. Additionally, the COVID-19 pandemic may have altered tobacco use behaviors among students, with increased stress, social isolation, and changes in academic routines potentially driving greater reliance on nicotine products [27]. This significant difference underscores the need for targeted interventions within dental education to address and mitigate the elevated risk of tobacco-related conditions and nicotine addiction among this specific demographic, which surpasses the overall risk of the Italian population. Fostering a health-conscious environment and aligning professional behavior with the principles of tobacco prevention and control in Italian dental settings are essential endeavors that can significantly contribute to the overall well-being of both dental practitioners and their patients, promoting a culture of health and preventive care within the dental community.

In our study, the smoking prevalence (30.4%) was lower than that reported in 2020 by Rodakowska et al*.* among Italian dental students (40.2%) [7], as well as that reported in 2021 by Kashmira et al. among Indian dental students (39.9%) [28]. This difference in smoking prevalence may be attributable to factors such as the impact of the COVID-19 pandemic, changes in tobacco pricing and taxation, the implementation of tobacco-related legislation, and demographic shifts in the Italian population. On the other hand, the smoking prevalence observed in our study was higher than that reported among dental students in Morocco (10%) [29], Japan (11.5%) [30], Iraq (10%) [31], Saudi Arabia (17%) [32], and Brazil (19%) [33]. In comparison with medical students from multicenter studies in Italy, the prevalence of smoking was significantly higher among dental students in our study (30.4% vs. 20.4%) [34]. This finding aligns with results from a global survey of health profession students, which reported that smoking prevalence among dental students in European countries was nearly twice that observed among medical students [35]. This difference may reflect variations in curricular emphasis, as medical students often receive more comprehensive training in general preventive medicine, including tobacco-related health risks, compared to dental students. In addition, as the data were collected in years different from our study (2023), variations in local socio-cultural contexts and country-specific tobacco control policies may have also contributed to the observed differences. This smoking prevalence in the current study (30.4%) was also higher than that reported in a meta-analysis for healthcare providers in Italy (21.4%) [36]. This higher prevalence among dental students compared to healthcare providers in general may be related to their younger age, exposure to academic and social stressors, and potential peer influence within the university environment, all of which are known risk factors for tobacco use.

This high prevalence of tobacco use among dental students has the potential to reduce the efficacy of healthcare providers as tobacco-cessation role models for their patients [14]. When it comes to health behaviors, providers are expected to serve as exemplary role models for their patients [14]. Tobacco usage conveys a conflicting message, and patients may be less inclined to follow the advice of a dental provider who engages in tobacco use [14]. Furthermore, a dental provider who uses tobacco may also find it unnecessary to educate their patients on tobacco prevention and cessation. Beyond students’ own tobacco use, smoking behaviors in their immediate social and professional environment may also shape attitudes towards tobacco control. Some dental and dental hygiene students have parents or other close relatives who work in dentistry or in the wider healthcare sector, and family members who smoke may implicitly convey that tobacco use is compatible with a professional career in health care. Similarly, observing faculty members or clinical supervisors who smoke on or around campus can undermine smoke-free norms and weaken the perceived importance of tobacco control. These relational influences may contribute both to the normalization of tobacco use and to the ambivalence towards tobacco-free policies observed in our sample.

In addition to the prevalence reported earlier, 27% of the dental students were current dual users. This was about 17% higher than that reported in Italy's general young adult population (10.0%) [37]. The higher proportion of dual use among dental students may be influenced by the concurrent availability and marketing of multiple tobacco products, which can encourage experimentation and sustained use. Moreover, the perception that alternating between products reduces health risks may contribute to the higher prevalence of dual use observed in this population compared to their peers in the general young adult population. This further reinforced the concerning engagement of surveyed dental students in tobacco product use, as about 4 in 10 dental students currently use at least one form of tobacco product.

We discovered that a significant proportion of students participating in our study were exposed to second-hand smoke (65.2%), ENDS aerosols (36.5%), and aerosols from other tobacco products (20.9%). While many dental and healthcare facilities have regulations in place prohibiting smoking and/or ENDS use in public areas and indoor spaces, these policies are often not rigorously enforced [7, 19]. This lack of enforcement was reported by 30% of the student, reflecting a noteworthy gap between the existence of regulations and their effective implementation within dental academic settings. It is then imperative for academic and healthcare institutions to enforce strict adherence to both outdoor and indoor anti-tobacco policies within school premises.

Tobacco use among health professional students has a negative impact on their beliefs and attitudes regarding tobacco prevention and control efforts [38]. Although the majority of students participating in our study supported tobacco control measures such as bans on selling tobacco to adolescents and advertisements, as well as restrictions on smoking in public places, more than 54% of them disagreed that healthcare providers using tobacco products are less likely to advise their patients to quit. As a result, it is becoming increasingly vital for dental education institutions in Italy to integrate tobacco prevention and cessation training opportunities into the curricula of the dental programs, but also offer tobacco treatment services not only for dental students but also for faculty, staff, and patients who use tobacco products or are nicotine addicts. A sizable proportion of smokers report having visited a dentist at least once in the previous year [39]. This suggests that dentists are in a good position to actively participate in public health activities targeted at reducing the health problems and mortality associated with tobacco use [40]. It is critical for the next generation of dentists to know the vital role they may play in implementing tobacco control efforts. However, dental students might underestimate their role in tobacco cessation efforts [41]. Factors that contribute to this underestimation include a training program that focuses more on the risks of tobacco than on practical counseling skills, limited opportunities for supervised counseling during clinical placements, and social norms that normalize smoking and ENDS use. Strengthening behavioral counseling competencies along with consistent role-modeling by faculty and clinical tutors will enhance students’ sense of responsibility and self-efficacy in providing cessation support.

This current study and findings from prior research conducted in India [42], Latin America [19], and 44 countries around the world [17] unequivocally highlight the deficiency in training dental students receive in delivering effective anti-tobacco cessation through both pharmacological and non-pharmacological approaches. Moreover, our study highlights a noticeable gap in the proportion of dental students who have undergone formal training in tobacco cessation approaches specifically tailored for patients using various forms of tobacco (cigarettes-68.7%, ENDS-38.3%, and other tobacco products-44.3%). This emphasizes the need for targeted educational initiatives to equip dental students with comprehensive skills for addressing the diverse landscape of tobacco products, which includes the rapid increase in ENDS use around the world [43], as well as to overcome the challenges associated with cessation in their patient population. Closing this training gap is essential to enhance the effectiveness of dental professionals in promoting tobacco prevention and cessation approaches in dental settings and ultimately improving public health outcomes in Italy.

The majority of students in our study (87.8%) reported awareness of an official school policy banning tobacco use, suggesting that such regulations are well established within the academic environment. However, only 72% of respondents perceived the policy to be enforced inside school buildings and clinical settings, highlighting a potential gap between the existence of regulations and their practical implementation. These findings underscore the importance of not only maintaining smoke-free campus policies but also ensuring consistent communication and rigorous enforcement, as policy effectiveness ultimately depends on student awareness and adherence.

Our findings have important implications for supporting the university’s existing smoke-free campus policy and the Law 584/1975 and Regional Law 17/2007 that prohibit smoking in healthcare facilities like the dental academic environment since 1975 [44–47]. The high prevalence of tobacco use, dual use, and exposure to secondhand smoke and ENDS aerosols reported by dental students suggests that the policy is not being fully enforced or adhered to within dental academic settings. Strengthening enforcement mechanisms, combined with educational campaigns that emphasize the health risks of tobacco and nicotine products, could help bridge this gap. Moreover, integrating targeted cessation support services for students, faculty, and staff would not only facilitate compliance with the smoke-free policy but also foster a healthier campus environment. By aligning these measures with our findings, the university can more effectively realize the goals of its smoke-free campus initiative while also positioning dental students as role models for tobacco prevention and cessation.

### Future directions

Overall, these results should be interpreted as reflecting the situation in a single public dental school in Southern Italy rather than providing nationally representative estimates. Nevertheless, they provide an informative case study of tobacco-related behaviors, exposures, and training gaps in this context, which may help generate hypotheses for future multi-center research on tobacco use and cessation training among dental students in Italy and other countries. A forthcoming initiative aims to expand the scope of data collection using the same survey methodology, with the intention of evaluating the prevalence of tobacco use and attitudes, as well as knowledge and practices related to cessation, across additional dental schools nationwide. This expansion aims to provide a more comprehensive understanding of the landscape of tobacco-related behaviors and cessation strategies within this European country’s broader dental education community. We also recommend that future studies conduct a detailed comparative analysis of dentistry and dental hygiene curricula to clarify how program-specific content may shape students’ knowledge, attitudes, and practices regarding tobacco use.

### Limitations and strengths

Our study has a few limitations. First, responses from the dental students were self-reported. Thus, there might be potential bias for underreporting of the prevalence of tobacco use and overreporting of the students' perception of tobacco prevention and control. Due to the convenience sampling nature of this single-site study, our findings might not be generalizable to all dental schools in Italy, as selection bias may have influenced the sample (e.g., more motivated or health-aware students). However, because we were able to sample 115 out of the 150 dental students in UPDS, our sample and findings are representative of UPDS. Another limitation was that our study did not capture information on the specific challenges faced by dental students in accessing or delivering tobacco cessation interventions, which may have provided further context for interpreting their perceptions and practices. A further limitation is that our analyses were primarily descriptive and relied on bivariate tests, which did not allow us to adjust for potential confounding factors and limits the causal interpretation of the observed associations. Finally, our study employs a cross-sectional design; therefore, it lacks longitudinal tracking, which limits insight into behavior change over time or the impact of the curriculum.

Despite these limitations, our study is the first Italian study to provide, in addition to cigarette smoking, the prevalence of current ENDS and other tobacco product use among dental students in Italy.

## Conclusion

The normalization of tobacco use, including ENDS products, among dental students engaged in our study is deeply concerning. This research endeavor marks a pivotal step toward fostering a healthier environment within the dental community and contributing to broader public health goals.

## Data Availability

The data supporting this research cannot be made available for privacy or other reasons.
